# Seasonal Alterations in Park Visitation, Amenity Use, and Physical Activity — Grand Forks, North Dakota, 2012–2013

**DOI:** 10.5888/pcd11.140175

**Published:** 2014-09-11

**Authors:** James N. Roemmich, LuAnn Johnson

**Affiliations:** Author Affiliations: LuAnn Johnson, Agricultural Research Service, US Department of Agriculture, Grand Forks, North Dakota.

## Abstract

**Introduction:**

Park amenities promote visitation and physical activity during summer. Physical activity declines during winter. Identifying park amenities that promote visitation during winter would increase year-round activity. The purpose of this study was to determine how park visitation, amenity choice, and physical activity intensity change across seasons.

**Methods:**

Physical activity intensity of children and adults was assessed at 16 parks in Grand Forks, North Dakota, during summer and fall of 2012, and winter and spring of 2013.

**Results:**

Park visitation was highest in spring and lowest in winter. Amenity use varied by season. Parks with water splash pads were visited more during summer, and playgrounds and open spaces were visited more during spring. Ice rinks were visited most in winter. Physical activity intensity was lowest in summer and highest in winter for each age group. The activity intensity observed for all young age groups ranged from 2.7 to 2.9 metabolic equivalents in summer and greater than 3 metabolic equivalents in all other seasons. Adults’ mean activity intensity was greater than 3 metabolic equivalents in winter.

**Conclusion:**

Information on park visitation, amenity use, and activity intensity across seasons is valuable; it can be used when designing or redesigning parks in order to promote year-round physical activity. Redesigning parks in cold climates to include ice rinks, sledding hills, cross-country skiing, and indoor areas for physical activity would increase winter visitation and allow the park to serve as a year-round resource for physical activity.

## Introduction

Participation in physical activity reduces the risk of many chronic diseases (1,2). Parks are an important community resource for promoting physical activity (3–6), which produces mental and physical health benefits (7–9). The choice to engage in physical activity at a park rather than to be sedentary is likely associated with several factors including sex, age, access to the park, and the amenities available at the park (10). The presence of certain park amenities such as playgrounds, sport courts, and paths seem to promote physical activity among park users (11–13). However, evidence regarding the most and least used amenities is inconsistent (12–14). Some of these inconsistencies could be explained by conducting studies in every season and in various geographical areas.

Traditionally, park-based physical activity is studied through observation during summer (13,15,16) and, occasionally, spring in warm climates (12). However, weather conditions probably influence park visitation and amenity use, even during the warm-weather seasons of spring and summer. Overall leisure-time physical activity declines during winter (17,18) when people must be willing to overcome cold weather and short days to engage in activity at a park. Providing amenities that promote physical activity during winter and making them accessible at neighborhood parks could increase activity during winter. To our knowledge, no studies have assessed park activities repeatedly during each of the 4 seasons to learn seasonal differences in visitation, amenity choice, and physical activity. The purpose of this study was to determine whether park visitation, amenity choice, and physical activity intensity of children, adolescents, and adults differ by season.

## Methods

During 2012–2013, physical activity type and intensity of park users was investigated at 16 parks in Grand Forks, North Dakota. The parks varied in size and amenities provided. This research was approved by the University of North Dakota Institutional Review Board.

Observers were trained in the use of the System for Observing Play and Recreation in Communities (SOPARC), which allows for measurement of the number of people of specified demographics (eg, age and sex) engaged in physical activities of sedentary, moderate, or vigorous intensity. Observers were trained on the use of SOPARC by using the standardized DVD and training materials (19), followed by several days of field training. Training continued until interobserver reliability reached at least 80% agreement with the trainer (J.N.R.) for demographics, number of people observed per scan, and activity intensity. Construct validity of SOPARC activity intensity has been established using heart rate as the criterion (20).

Park areas were sectioned into component parts to ease scoring when many users were present and to allow for analysis by amenity. Systematic observations were made during summer (July 17–August 18) and fall (September 29–October 29) of 2012, and winter (January 2–February 9) and spring (May 7–June 8) of 2013. Systematic observations of the target areas took place at 3 intervals (10:30–11:30 am, 2:30–3:30 pm, and 5:30–6:30 pm) each day for 3 weekdays and 1 Saturday during each of the 4 seasons. Observers performed a rapid visual scan to determine the number of children and adults and their sex, age category (young child 0–5 y, child 6–12 y, teen 13–18 y, and adult ≥19 y), activity intensity (sitting, standing, walking/moderate, or vigorous), and activity location (eg, at the swings or slides). Intensity was scored at the moment of observation and not by the general activity. Activity level classifications were converted to metabolic equivalent (MET) intensities (sitting = 1.25 METs; standing = 1.5 METs; moderate = 3.0 METs; vigorous = 6.0 METs) based on described values (19,21,22). Observers did not interact with the patrons, except to acknowledge and return perfunctory greetings. Park visitors were not informed of the research as this might elicit a change in behavior. Heat index or wind chill according to weather history at wunderground.com were recorded. The mean (SE) heat index or wind chill over observation days were 26.6°C (5.0) in summer, 8.2°C (9.3) in fall, −12.8°C (6.0) in winter, and 20.2°C (3.9) in spring. Observations were rescheduled when weather dictated (eg, raining, real temperature of less than −15°C).

Ice sheets (outdoor hockey rinks and ice rinks for skating), sand volleyball courts, basketball courts, and tennis courts were all categorized as “sport courts”; baseball fields, soccer fields, and football fields were “sport fields.” SOPARC data were aggregated to determine number of users and activity intensity according to sociodemographic characteristics (eg, age and sex). Data are reported as means (SEs). For each age category, a log-linear model was used to test whether the number of park visitors differed by sex, by season, or by an interaction of sex and season. For each park amenity, a log-linear model was used to test whether the number of visitors differed by season. The Glimmix procedure in SAS version 9.3 (SAS Institute, Inc) was used to fit the log-linear models by specifying the Poisson distribution and χ^2^ statistics. Two-way analysis of variance was used to test for the effects of sex, season, and the interaction of sex and season on activity intensity. Tukey contrasts were used for post-hoc comparisons. SAS version 9.3 was used for all analyses.

## Results

Children’s park visitation differed by season with significantly greater visits in summer (678 children observed), followed by spring (512), fall (260), and winter (108). Significantly more young children who visited the park were boys than girls (Table 1). More boys than girls aged 6*–*12 y visited parks in spring and winter. More adolescent boys than girls (aged 13*–*18 y) were observed in the fall and winter, but more girls than boys in the spring. More adult men than women were observed during fall and winter. 

**Table 1:** Number of Visitors at Parks by Age Group, Sex, and Season — Grand Forks, North Dakota, 2012 and 2013^a^

Age, y	Summer 2012		Fall 2012		Winter 2013		Spring 2013	
	Male	Female	Male	Female	Male	Female	Male	Female
0–5^b^	373	305	136	124	59	49	277	235
6–12^c^	294^d^	290^d^	479^e^	445^e^	194^f^	105^g^	802^h^	614^i^
13–18^c^	162^d^^,^ e	175^d^^,^ e	219^e^	71^f^	204^d^^,^ e	60^f^	151^d^	209^e^
≥19^c^	848^d^^,^ e	972^d^^,^ f	774^e^	655^g^	332^h^	165^i^	1,055^f^	978^f^

a Seasons were summer and fall of 2012 and winter and spring of 2013. Data are the number of individuals tallied at all 16 study parks over 12 observations (3 times per day on 3 weekdays and 1 weekend day).

b From log-linear models, season main effect *P* < .001; each season differs from all others for both males and females.

c From log-linear models, like letters (d,e,f,g,h,i) within same row are not significantly different by Tukey contrasts.

We determined the numbers of park visitors using each amenity (Table 2). As a result of ice sheets being included in the “courts” category, courts were visited most in winter and then spring. Playgrounds, a Frisbee golf course, and open spaces were visited most in the spring and then summer. Splash pads, gardens, and shelters were visited most in summer. Walking path use was greatest in spring and summer, followed by fall, and then winter. Sport fields were used most often in fall, followed by spring and then summer. 

**Table 2:** Number of Visitors Using Park Amenities, by Season^a^ — Grand Forks, North Dakota, 2012 and 2013

Amenity^b^	No. of Parks With the Amenity	Season			
		Summer 2012	Fall 2012	Winter 2013	Spring 2013
Sport courts	Hockey, 11	146^c^	74^d^	668^e^	281^f^
	Basketball, 12				
	Volleyball, 2				
	Tennis, 3				
Playgrounds	16	1024^c^	700^d^	213^e^	1,624^f^
Frisbee golf	1	156^c^	147^c^	6^e^	268^d^
Open spaces	11	106^c^	60^d^	71^d^	158^e^
Splash pads	2	195^c^	8^d^	0	36^e^
Gardens	2	112^c^	21^d^	0	44^e^
Shelters	11	584^c^	183^d^	0	41^e^
Walking paths	7	583^c^	444^d^	115^e^	665^c^
Sport fields	Baseball, 11	152^c^	716^d^	0	499^e^
	Soccer, 7				
	Football, 3				
Treed areas	8	88^c^	57^c^	24^d^	70^c^

a Data are the number of individuals tallied at all 16 study parks over 12 observations (3 times per day on 3 weekdays and 1 weekend day).

b From log-linear models, like letters (c,d,e,f) in the same row are not significantly different by Tukey contrasts (all *P* values are < .001).

We collected data on intensity of physical activity (Table 3). The intensity of young children’s physical activity at the park differed significantly by season. Young children were most intensely active during winter and least active in summer. Similarly the activity of children aged 6 to 12 years was most intense during winter and least intense during summer. Boys were more intensely active than girls. The interaction of sex and season for activity intensity of adolescents (aged 13–18 y) was significant. Adolescent boys were more intensely active than girls during winter and were more intensely active in winter than other seasons. Adolescent girls’ activity intensity was greater in fall than summer. 

**Table 3:** Activity Intensity (METs) in Parks by Age Group, Sex, and Season — Grand Forks, North Dakota, 2012 and 2013^a^

Age, y	Summer 2012		Fall 2012		Winter 2013		Spring 2013	
	Male	Female	Male	Female	Male	Female	Male	Female
0–5^b^	2.9 (0.1)	2.7 (0.1)	3.4 (0.1)	3.2 (0.2)	4.6 (0.2)	4.5 (0.2)	3.6 (0.1)	3.4 (0.1)
6–12^b^	2.9 (0.1)	2.7 (0.1)	3.6 (0.1)	3.4 (0.1)	4.8 (0.1)	4.3 (0.2)	3.5 (0.1)	3.4 (0.1)
13–18^c^	2.9 (0.1)^d^	2.7 (0.1)^d^	3.4 (0.1)^e^	3.6 (0.2)^e^	4.5 (0.1)^f^	3.3 (0.2)^d^^,^ e	3.3 (0.1)^d^^,^ e	3.1 (0.1)^d^^,^ e
≥19^c^	2.7 (0.1)^d^	2.2 (0.1)^e^	2.5 (0.1)^f^	2.2 (0.1)^e^^,^ g g	4.2 (0.1)^h^	3.2 (0.1)^i^	2.8 (0.1)^d^	2.4 (0.1)^f^^,^ g

Abbreviation: METs, metabolic equivalents.

a Data are the mean (standard error) METs of people assessed in 16 parks observed 3 times per day on 3 weekdays and 1 weekend day.

b From two-way analysis of variance, season main effect is significant: winter > spring; fall > summer.

c From two-way analysis of variance, like letters (d,e,f,g,h,i) in the same row are not significantly different by Tukey contrasts.

The interaction of season and sex was also significant for the intensity of adults’ physical activity. Men were more active than women in all seasons. Men had greater activity intensity in winter (4.2 METs) than in all other seasons. The intensity of men’s activity in spring and summer (*P* = .005) was greater than in fall (2.5 METs). Women also had greater activity intensity in winter (3.2 METs) than in all other seasons. Women’s activity intensity was greater in spring (*P* = .01) than in summer.

## Discussion

This study extends this area of research by providing initial evidence of seasonal effects on the visitation of parks, amenity use, and physical activity intensity of park visitors. Visitation was highest during spring and could be attributed to the advent of warmer weather. These results raise questions regarding the generalizability of results from park observation studies that occur during spring or summer, because seasonal differences in both activity intensity and park visitation can be large. In northern climates, assessments conducted during spring or summer would provide different estimates of park visitation, amenity use, and the importance of parks as community resources for physical activity than assessments conducted during spring and fall.

Total leisure-time physical activity is lower in the winter than other seasons (23,24). Moreover, obesity prevalence among young people is greatest in winter and lowest in spring and summer (25); thus, park visitation should be promoted, especially during the winter. Understandably, the lowest park visitation was during the winter. The outdoor temperatures and wind chill factors during a northern plains winter are not as comfortable as during the spring and summer. Additional promotion of winter activity by park designers, park boards, and park managers could increase winter visitation. Sport fields are available during the winter season for active pursuits such as fat-tire bicycling, snowshoeing, or cross-country skiing, but few visits were observed to these areas. During the winter, nets for volleyball and tennis are removed from sport courts and ice sheets are added for hockey and ice skating. Unlike other age groups who visited the park less in the winter, adolescent boys visited at similar rates during cold weather to play hockey.

Future research should determine which park amenities and programs promote winter park visitation and physical activity by other demographic groups. Perhaps sledding hills could draw young and middle-school–aged children with their parents. Indoor fitness centers are popular in Grand Forks. Perhaps, in a northern climate where snow is common, park visitation and activity could be increased by adding smaller indoor facilities for yoga, dance, climbing, table tennis, volleyball, and martial arts to neighborhood parks. 

The parks observed in our study had fewer programs in winter than in summer. Programming outdoor activities such as ice skating lessons, outdoor ice hockey leagues, cross-country skiing lessons and group sessions on groomed trails, and winter walking and running groups could increase active park use in winter. Flowers in bloom, the textures and colors of the leaves of shrubs, and the changing colors of foliage encourage park visitation during the warmer months. In contrast, parks lack such natural ornamentation during winter. Perhaps winter programs to create snow sculptures or holiday ornamentation would draw children and adults to visit. Park infrastructure such as warming huts and lighting may also increase visitation during the short days of winter, as safety is an important factor in one’s decision to go to a park (26).

Although park visits are lower during winter, the activity intensity is greater than 3 METs, which is considered moderate-to-vigorous physical activity (MVPA) and associated with health benefits such as lower adiposity and lower risk for metabolic syndrome (27). Park visitors were more intensely active during winter than in any other season, largely because of ice skating activities and low temperatures that encourage motor movement. Consistent with previous research findings, the lowest activity intensity was recorded during summer (28). Perhaps people are least intensely active outdoors when the temperatures are high.

Having winter amenities in parks would promote winter park use, and providing such amenities should be a goal of park design or redesign. Visits were highest during spring, and activity intensity was lowest during summer. Seasonal differences in the number of visits, amenity use, and activity intensity, even during warm seasons, suggests that results collected during one season are not generalizable to all seasons. Our study provides insights into the amenities that draw individuals to parks during each season. Similar research is needed in other geographical areas with a different climate, such as the desert, where the summer weather may be more of a challenge than winter weather to increasing park visits and physical activity. Information on the number of park visits, extent of amenity use, and the activity intensity of those who use the park during each season is extremely useful for those engaged in designing or renovating parks to promote year-round physical activity.
